# Inhibition of the SET8 Pathway Ameliorates Lung Fibrosis Even Through Fibroblast Dedifferentiation

**DOI:** 10.3389/fmolb.2020.00192

**Published:** 2020-08-05

**Authors:** Keita Ugai, Shuichi Matsuda, Hideki Mikami, Ayako Shimada, Tomoko Misawa, Hiroyuki Nakamura, Koichiro Tatsumi, Masahiko Hatano, Toshihiko Murayama, Yoshitoshi Kasuya

**Affiliations:** ^1^Department of Biomedical Science, Graduate School of Medicine, Chiba University, Chiba, Japan; ^2^Laboratory of Chemical Pharmacology, Graduate School of Pharmaceutical Sciences, Chiba, Japan; ^3^Department of Respirology, Graduate School of Medicine, Chiba University, Chiba, Japan; ^4^Department of Biochemistry and Molecular Pharmacology, Graduate School of Medicine, Chiba University, Chiba, Japan

**Keywords:** idiopathic pulmonary fibrosis, myofibroblast, dedifferentiation, SET8, UNC0379

## Abstract

Idiopathic pulmonary fibrosis (IPF) is a fatal lung disease of unknown etiopathogenesis. The activation of extracellular matrix (ECM)-producing myofibroblasts plays a key role in fibrotic tissue remodeling. The dedifferentiation of myofibroblasts has attracted considerable attention as a promising target for the development of effective therapeutic interventions against IPF. Here, we screened a small library of epigenetics-related inhibitors using dedifferentiation assay of lung myofibroblasts prepared from a patient at the terminal stages of IPF and chose UNC0379. The inhibition of SET8, a histone H4 lysine 20 (H4K20) monomethyltransferase, by UNC0379 markedly suppressed the expression of α-smooth muscle actin (SMA) and ED-A-fibronectin in myofibroblasts. In IPF myofibroblasts, SET8 expression and H4K20 monomethylation (H4K20me1) levels, which were significantly higher than those in normal human lung fibroblasts, were reduced upon treatment with UNC0379. Hence, the changes in the expression of the two fibrotic markers clearly correlated with those in SET8 expression and H4K20me1 level. Furthermore, in a mouse model of bleomycin (BLM)-induced lung fibrosis, the intratracheal administration of UNC0379 at an early fibrotic stage markedly ameliorated the histopathological changes associated with collagen deposition in the lungs. However, treatment with UNC0379 did not significantly affect the number of proinflammatory cells or cytokine production in the bronchoalveolar lavage fluids from mice treated with BLM. In the BLM-injured lung, SET8 was predominantly localized to the nuclei of α-SMA-positive cells, which colocalized with H4K20me1. Taken together, our results indicate that the inhibition of SET8 resulting in myofibroblast dedifferentiation may partly mitigate lung fibrosis without affecting the inflammatory responses.

## Introduction

Idiopathic pulmonary fibrosis (IPF) is one of the most common causes of interstitial pneumonia, characterized by progressive and irreversible fibrotic scar formation, resulting in lung malfunction. Patients with IPF show a poor prognosis with a median survival of 3–5 years and have an increased risk of lung cancer and pulmonary hypertension, making IPF one of the most devastating lung diseases (Wynn and Ramalingam, 2012; Lederer and Martinez, 2018). The mortality of patients with IPF is correlated with the extent of fibrotic focus formation, which results from the abnormal and excessive accumulation of extracellular matrix (ECM) components, including collagen, fibronectin (FN), and elastin. Hence, myofibroblasts that produce excess ECM play a crucial role in fibrotic tissue remodeling.

Myofibroblasts differentiate from various cell types under certain pathological conditions, including fibroblasts (known as fibroblast to myofibroblast differentiation, FMD), epithelial cells (known as epithelial-mesenchymal transition, EMT), endothelial cells (known as endothelial-mesenchymal transition, EndoMT), fibrocytes, and pericytes (Hong et al., 2007; Rock et al., 2011; Kendall and Feghali-Bostwick, 2014; Tsukui et al., 2015; Suzuki et al., 2017). Among these, FMD is well known as the main route of myofibroblast establishment (Tanjore et al., 2009; Tsukui et al., 2015). Therefore, the efficient inhibition of FMD may lead to the development of therapeutic options for IPF. However, most patients with IPF who present subjective symptoms, have already progressed into the early fibrotic stage due to reduced forced vital capacity (King et al., 2001). Hence, resolving established fibrosis through the promotion of myofibroblast dedifferentiation has great potential as a beneficial intervention in patients with IPF (Rangarajan et al., 2018). Monitoring the dedifferentiation of TGF-β1-induced myofibroblasts represents a useful assay system to screen antifibrotic agents (Sieber et al., 2018). However, prostaglandin E2, which can dedifferentiate TGF-β1-induced myofibroblasts, fails to revert the phenotype of myofibroblasts from the lungs of patients with IPF (Huang et al., 2010; Garrison et al., 2013). These findings suggest that using IPF lung-derived myofibroblasts is more relevant when screening for antifibrotic agents. In this context, we prepared myofibroblasts from an end-stage IPF/usual interstitial pneumonia (UIP) patient to screen for agents capable of dedifferentiating myofibroblasts.

The epigenetics involved in the pathogenesis of IPF has been thoroughly investigated. Some key findings of these studies include: (1) a DNA hypermethylation-associated decrease in mRNA expression was detected for 16 genes in patients with IPF (Sanders et al., 2012) using DNA methylation array and RNA expression microarrays; (2) spiruchostatin A, a histone deacetylase blocker, efficiently reduced the uncontrolled proliferation of IPF fibroblasts (Davies et al., 2012); (3) lysine-specific demethylase 1 contributes to FMD and fibrosis through the TGF-β1/Smad3 signaling pathway (Pan et al., 2020); (4) among non-coding RNAs, miRNAs function as both profibrotic and antifibrotic mediators in IPF (Tzouvelekis and Kaminski, 2015; Rubio et al., 2020). On the other hand, the long non-coding RNA activated by TGF-β promotes EMT during pulmonary fibrosis by competitively binding miR-200c (Liu et al., 2018). Furthermore, a long intergenic non-coding RNA, linc01140, which is upregulated in IPF-derived fibroblasts, has been implicated in cellular proliferation and inflammatory responses (Hadjicharalambous et al., 2019). Hence, the screening of agents able to modulate the epigenetic characteristics of the IPF myofibroblasts is of great therapeutic interest.

In this study, a small library of epigenetics-related inhibitors was screened using our IPF-myofibroblast dedifferentiation assay. UNC0379, a specific inhibitor of SET8 (a histone H4 lysine 20 (H4K20) monomethyltransferase, also known as SETD8, PR-SET7, and KMT5A) was identified as an efficient dedifferentiating agent for IPF myofibroblasts. Recent reports have demonstrated that SET8 is a key player in maintaining the proliferation, cell survival, and metastatic behavior of tumor cells. Hence, SET8 is expected to be a potential target in malignant tumors, including medulloblastoma, hepatocellular carcinoma, and high-risk neuroblastoma (Veo et al., 2019; Wu et al., 2020; Veschi et al., 2017). We also demonstrated that mice treated with UNC0379 at an early fibrotic stage showed a marked alleviation of bleomycin (BLM)-induced lung fibrosis. Therefore, inhibition of SET8 may represent a new therapeutic strategy not only for tumors but also for pulmonary fibrosis.

## Materials and Methods

### Cell Culture

Lung tissue from a 46-year-old male patient who underwent lung transplantation due to severe IPF (UIP pattern) was obtained after surgery. The patient provided informed consent and the study was approved by the Ethics Committee of the Chiba University, Graduate School of Medicine and Hospital. The fibrotic lung tissue was minced into small pieces and incubated in Dulbecco’s modified Eagle’s medium (DMEM) supplemented with 1 mg/ml collagenase type I (Worthington, Lakewood, NJ, United States), 0.5 mg/ml dispase (Thermo Fisher Scientific, Waltham, MA, United States), 2 U/ml DNase (Qiagen, Valencia, CA, United States), 0.1 mg/ml streptomycin, and 100 U/ml penicillin at 37°C for 15 min with gentle shaking. After washing with DMEM, the pieces were transferred to a 90 mm culture dish (IWAKI Science Products, Shizuoka, Japan) and immersed in DMEM supplemented with 15% fetal bovine serum (FBS), the antibiotics described above, and cultured at 37°C with 5% CO_2_. The cell growth was monitored every day, and the culture medium was changed every 4 days. When the dish reached confluence (after about 14 days), the cells were harvested as cells at passage 0. Subsequently, the expanded cells at passage 3 were subjected to flow cytometry, and the IPF lung-derived fibroblasts were denoted as IPF myofibroblasts (IPF-MyoF). Additional IPF myofibroblasts (DHLF, diseased human lung fibroblasts (IPF), CC-7231) and normal human lung fibroblasts (NHLF) were purchased from Lonza Bioscience (Basel, Switzerland). In addition, myofibroblasts derived from a patient with pleuroparenchymal fibroelastosis, termed 19-YM in our previous study, were also used (Yamazaki et al., 2017). All primary fibroblasts were maintained in 15% FBS/DMEM and used for the experiments at passage 6–8.

### Western Blotting (WB)

The cells were plated in 6-well plates (IWAKI) and maintained in DMEM containing 15% FBS overnight. After adding fresh medium, the cells (6 × 10^5^ cells/well) were treated with 10 ng/ml human recombinant TGF-β1 (PeproTech, Rocky Hill, NJ, United States), 10 μM UNC0379 (Cayman Chemical, Ann Arbor, MI, United States) or TGF-β1 plus UNC0379 for 48 h. The cells were then lysed and subjected to WB with mouse anti-α-SMA monoclonal antibody (Ab) (ab7817; Abcam, Cambridge, United Kingdom), mouse anti-ED-A-FN monoclonal Ab (sc-59825, clone IST-9, ED-A domain-specific; Santa Cruz Biotechnology, Dallas, TX, United States), rabbit anti-SET8 monoclonal Ab (#2996; Cell Signaling Technology, Danvers, MA), mouse anti-H4K20me1 monoclonal Ab (C15200147; Diagenode, Liege, Belgium), or mouse anti-β-actin monoclonal Ab (A5441, clone AC-15; Sigma-Aldrich, St. Louis, MO, United States) (1:1000 dilution) followed by incubation with horseradish peroxidase-conjugated anti-mouse or anti-rabbit IgG (1:2000 dilution). The blot was exposed to an X-ray film (FUJIFILM, Tokyo, Japan) to visualize the immunoreactive signals detected by chemiluminescence. The intensity of the signals on the developed X-ray films was quantified using the ImageJ software (National Institutes of Health, Bethesda, MD, United States).

### Immunofluorescence

As described above, the cells were plated on 8-well chamber slides (Thermo Fisher Scientific) (2 × 10^4^ cells/well) and treated with the corresponding agents before being subjected to immunofluorescence analysis. The cells were incubated with the primary Ab, anti-α-SMA Ab, or anti-ED-A-FN Ab (1:200 dilution), followed by staining with a secondary antibody, namely Alexa-594-conjugated anti-mouse IgG (Thermo Fisher Scientific) (1:400 dilution). To visualize the morphology, the cells were counterstained with Alexa-488-conjugated phalloidin (Thermo Fisher Scientific). To obtain lung sections, mice were sacrificed 14 days post-instillation (dpi) of BLM (after intratracheal administration of phosphate-buffered saline (PBS) or UNC0379 at 7, 8, and 9 dpi) or PBS. The lung lobes were fixed, dehydrated, and frozen. Freshly cut lung sections (5 μm thick) were placed on adhesive glass slides (APS-01, Matsunami Glass Ind., Osaka, Japan) and pretreated with 1:10 FcR blocking agent (Miltenyi Biotech, Gladbach, Germany) for 10 min. The samples were then treated with various Abs, namely anti-SET8 Ab, anti-α-SMA Ab, goat anti-prosurfactant protein-C (SFPC) polyclonal Ab (sc-7706; Santa Cruz Biotech, Santa Cruz, CA), mouse anti-S100A4 monoclonal Ab (ab93283; Abcam) or anti-H4K20me1 Ab (1:100 dilution), followed by staining with appropriate fluorescein-conjugated second Ab (1:200 dilution), and 4’,6-diamidino-2-phenylindole (DAPI) was used for nuclear staining. The stained cells and sections were observed under a fluorescence microscope (Axio Imager A2; Zeiss, Oberkochen, Germany).

### Mice

Male C57BL/6J mice were purchased from Clea Japan (Tokyo, Japan). The animals were housed under specific pathogen-free conditions with free access to water and food in the Animal Resource Facility of Chiba University and cared for according to the animal care guidelines of Chiba University. The studies were conducted according to a protocol approved by the Animal Welfare Committee of Chiba University.

### BLM-Induced Lung Fibrosis Model

Mice (10–12 weeks old) were anesthetized and subjected to a single intratracheal instillation of BLM hydrochloride (3 mg/kg; Nippon Kayaku, Tokyo, Japan) dissolved in PBS using a Microsprayer atomizer (PennCentury, Philadelphia, PA, United States). The control mice were administered sham treatment with PBS on days 0, 7, 8, and 9 (PBS + PBS: PBS group). To evaluate the effect of UNC0379 on BLM-induced fibrosis, UNC0379 (1 mg/kg/day) or PBS (sham treatment) was intratracheally administered to anesthetized mice at 7, 8, and 9 dpi of BLM (BLM + PBS: BLM group or BLM + UNC0379: BLM-UNC0379 group). Six mice from each group were used in the following analyses.

To evaluate the histopathological changes in the lung samples at 14 dpi of BLM, freshly cut lung sections (5-μm thick) were placed on adhesive glass slides and stained with hematoxylin-eosin (HE). To visualize the fibrotic lesions, staining with Masson’s trichrome (MT) or picrosirius red (PR) was performed. The changes in the fibrotic lung samples were evaluated semi-quantitatively using a modified Ashcroft method with a scoring grade of 0 to 8 (Hübner et al., 2008). In addition, the collagen content of the left lung was measured using Sicol Soluble Collagen Assay Kit (Biocolor Life Science Assays, Carrickfergus, United Kingdom) according to the manufacturer’s protocol.

### Evaluation of Cell Populations and Cytokines in Bronchoalveolar Lavage (BAL) Fluids

Four mice from each experimental group (PBS, BLM, and BLM-UNC0379) were used in a cytokine array. At 10 dpi of PBS or BLM, the trachea was exposed and lavaged three times with 1.2 ml ice-cold PBS using a 20-gauge catheter. After centrifuging the BAL fluid at 400 × *g* for 5 min, the resulting supernatants were stored at −80°C as samples for use in the cytokine array. The cell pellets were resuspended in PBS and stained with Diff-Quick (Sysmex Co., Kobe, Japan) for cell counting using a hemocytometer. For the cytokine array, the BAL fluids from two mice form each group were collected, mixed, and subjected to Proteome Profiler Mouse Cytokine Array Kit for 40 cytokines (ARY006; R&D systems, Inc., Minneapolis, MN). The WB cytokine array was performed twice according to the manufacturer’s instructions. The resulting signals for each cytokine were normalizing to the positive internal control included in the array membrane and evaluated using an Image Gauge V4.21 (FUJIFILM).

### Statistical Analysis

Data are expressed as the mean ± standard error of the mean (SEM). Statistical analysis was conducted using GraphPad Prism version 6 (GraphPad Software, San Diego, CA, United States). The statistical significance was determined using analysis of variance (ANOVA) followed by Tukey’s test. A *p*-value of < 0.05 was considered to be significant.

## Results and Discussion

### UNC0379, a SET8 Inhibitor Dedifferentiates IPF-MyoF

**FIGURE 1:**
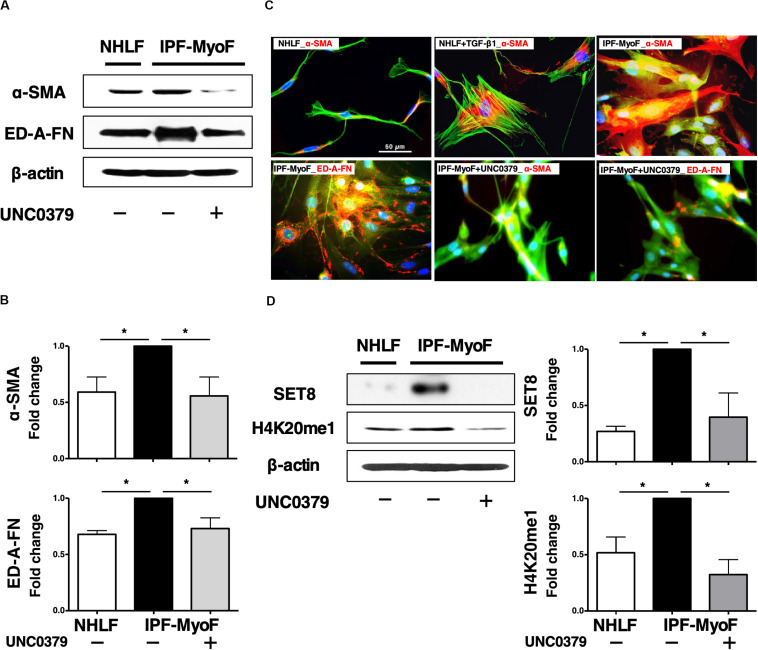
UNC0379 reversed the phenotype of IPF-MyoF to NHLF. **(A)** IPF-MyoF and NHLF were treated with vehicle (PBS) or 10 μM UNC0379 for 48 h. The expression of α-SMA, ED-A-FN, and β-actin (internal control for normalization) in the cell lysates were analyzed by WB. **(B)** Relative quantification data of the levels of α-SMA and ED-A-FN are expressed as fold changes with respect to the control value of IPF-MyoF with the vehicle. **(C)** Representative immunofluorescence staining images of α-SMA and ED-A-FN (red) in IPF-MyoF treated with or without UNC0379 and NHLF treated with or without 10 ng/ml TGF-β1. The actin cytoskeleton and the nuclei were counterstained with phalloidin (green) and DAPI (blue), respectively. **(D)** The expression of SET8 and H4K20me1 in IPF-MyoF and NHLF treated as in **(A)** were analyzed by WB. All quantitative data are shown as the mean ± SEM (*n* = 3). *p < 0.05 (measured by one-way ANOVA followed by Tukey’s test).

As shown in Supplementary Figure S1, we confirmed that ≥ 90% of the IPF-MyoF were α-SMA^+^ED-A-fibronectin (FN)^+^, which have been well-characterized as upregulated proteins in FMD (Kendall and Feghali-Bostwick, 2014; Yamazaki et al., 2017). On the other hand, > 90% of NHLF were positive for S100 calcium binding protein A4 (S100A4, also known as fibroblast-specific protein 1), while the IPF-MyoF showed negligible S100A4 expression. Then, we screened a small library of epigenetics-related inhibitors using the IPF-MyoF dedifferentiation assay with monitoring downregulation of α-SMA and ED-A-FN as indices. As illustrated in Supplementary Figure S2, ten compounds were chosen from a small library of 80 epigenetics-related inhibitors as candidates for the IPF-MyoF screening system (Sigma-Aldrich) after a PubMed search. After the first and second rounds of screening and reproducibility analysis, UNC0379 was chosen as a potential agent for the dedifferentiation of IPF-MyoF. In preliminary studies, we evaluated the dose of UNC0379 needed to dedifferentiate myofibroblasts without cell death, since the inhibition of SET8 by relatively high doses of UNC0379 occasionally induces cellular senescence and caspase activity (Tanaka et al., 2017; Veschi et al., 2017). Although the MTT assay revealed that treatment of subconfluent IPF-MyoF with 10 μM UNC0379 for 48 h reduced the cell viability by less than 15%, there was no significant difference between the treated and untreated/control group. As shown in Figures 1A,B, the expression of α-SMA and ED-A-FN was significantly higher in IPF-MyoF compared to those in NHLF, which were reversed by the treatment of IPF-MyoF with UNC0379.

The elongated and spindle-shaped fibroblasts change into a flat and polygonal shape upon TGF-β1-induced FMD, which is accompanied by the formation of α-SMA-based contractile actin bundles and maturation of focal adhesions (Sampson et al., 2011; Sandbo and Dulin, 2011). Hence, the morphological dynamics of fibroblasts reflect their state of cellular differentiation and dedifferentiation. As shown in Figure 1C, TGF-β1 induced a drastic morphological change in NHLF, from a thin fibrous shape to an expanded polygonal shape, which was associated with an increase in α-SMA. In case of IPF-MyoF, their morphology was different from that of untreated NHLF but similar to that of TGF-β1-treated NHLF. Likewise, high expression of α-SMA throughout the cell body and marked perinuclear accumulation of ED-A-FN were observed. Application of UNC0379 to the IPF-MyoF resulted in a spindle-shaped morphology accompanied by a decrease in the expression of both α-SMA and ED-A-FN. These findings were supported by the results of the flowcytometric analysis (Supplementary Figure S3). α-SMA^+^ED-A-FN^+^ IPF-MyoF had high SSC that could be proportional to intracellular organelle complexity, which was significantly reduced by the treatment of IPF-MyoF with UNC0379. In conjunction with this phenomenon, the SSC^low^FSC^low^ cell population mostly comprised of α-SMA^–^ED-A-FN^–^ and α-SMA^low^ED-A-FN^–^ fractions were negligible in the IPF-MyoF but significantly increased in response to the treatment with UNC0379. Along with the WB analysis, these results suggest that UNC0379 can induce the dedifferentiation of IPF-MyoF based on specific marker expression and morphology.

Next, we profiled the SET8/H4K20 monomethylation signaling axis, the target of UNC0379 in IPF-MyoF. As shown in Figure 1D, both SET8 expression and H4K20 monomethylation in the IPF-MyoF were significantly higher than those in NHLF, indicating that SET8 signaling may be activated in pathologically differentiated fibroblasts. Treatment of IPF-MyoF with UNC0379 reversed SET8 expression and H4K20 monomethylation to the levels observed in NHLF, similar to the results obtained with α-SMA and ED-A-FN. Therefore, the question arises whether the application of UNC0379 to the cells results in the downregulation of SET8 via a protein degradation system elicited by UNC0379 (Wang et al., 2015). However, the concomitant application of UNC0379 and MG132, a proteasome inhibitor, did not affect the downregulation of SET8 (data not shown). Recently, another study found that UNC0379 treatment had an effect similar to SET8 knockdown in hepatic carcinoma cell lines (Wu et al., 2020). Therefore, the UNC0379-induced downregulation of SET8 may occur in various cell types, including IPF-MyoF. Further study of the mechanisms underlying inhibitor-induced downregulation of the target molecule is needed, given that UNC0379 is a selective, substrate-competitive inhibitor of SET8 (Ma et al., 2014).

Tissue and cellular heterogeneity at the genomic and epigenetic levels, in pulmonary fibrosis including IPF, is a widely accepted concept (Tzouvelekis and Kaminski, 2015; Habiel and Hogaboam, 2017; Yu et al., 2018). This may translate to heterogeneity in terms of the effectiveness of treatment. To elucidate whether UNC0379 can universally dedifferentiate lung myofibroblasts derived from different origins, DHLF and 19Y-M were also analyzed (Yamazaki et al., 2017). In addition to the dedifferentiation assay, the effect of UNC0379 on the TGF-β1-mediated further differentiation of IPF-MyoF was also evaluated, because the proto-myofibroblasts, an intermediate state between fibroblasts and fully differentiated myofibroblasts, may be included in those lung fibroblasts to some extent (Hinz, 2007). As shown in Supplementary Figure S4, the levels of α-SMA, ED-A-FN, and SET8 in IPF-MyoF were significantly upregulated in response to TGF-β1, which was suppressed in the presence of UNC0379. Hence, the changes in the expression of the two fibrotic markers correlated with that of SET8. Moreover, in DHLF and 19Y-M, UNC0379 downregulated α-SMA, ED-A-FN, and SET8. A positive correlation was observed between changes in the expression of the three molecules under each experimental condition, although a significant difference was not detected in terms of α-SMA expression in DHLF and SET8 expression in 19Y-M. These results suggest that UNC0379 can induce myofibroblast dedifferentiation and inhibit additional FMD in at least three primary cultured myofibroblast lines with different clinical backgrounds. In NHLF, on the other hand, α-SMA and ED-A-FN were significantly upregulated in response to TGF-β1, although SET8 was marginally upregulated by TGF-β1 despite its low basal levels. However, the upregulation of ED-A-FN was sensitive to UNC0379, suggesting that SET8 may be involved in TGF-β1-induced FMD in normal fibroblasts. The characterization of the precise molecular mechanisms underlying myofibroblast dedifferentiation induced by the inhibition of the SET8/H4K20me1 axis is an important topic for future studies. After confirming the *in vitro* antifibrotic effect of UNC0379, we next investigated the effect of UNC0379 in the BLM-induced lung fibrosis model.

### UNC0379 Ameliorates Lung Fibrosis Without Affecting the Inflammatory Response

**FIGURE 2:**
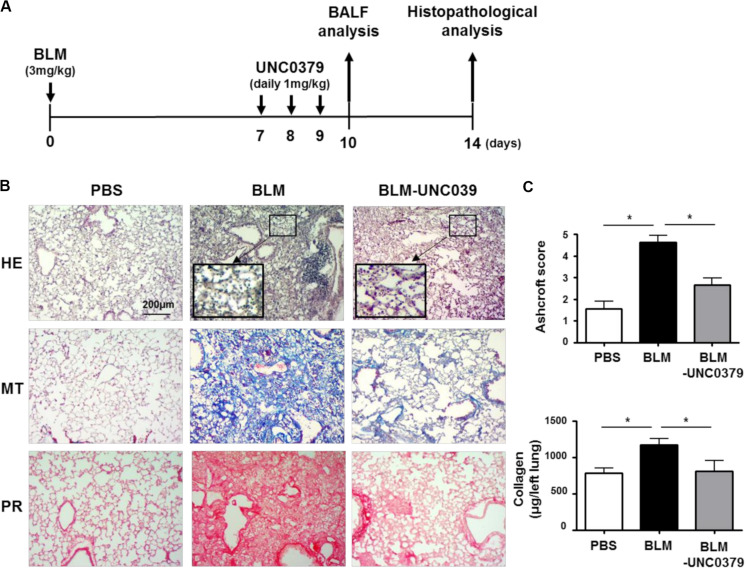
UNC0379 attenuated BLM-induced lung fibrosis. **(A)** Timeline of drug administration and analysis. Mice were intratracheally administered PBS on days 0, 7, 8, and 9 (PBS group), BLM on day 0 and PBS on days 7, 8, and 9 (BLM group), and BLM on day 0 and UNC0379 (1 mg/kg) on days 7, 8, and 9 (BLM-UNC0379 group). The BAL fluids were recovered at 10 dpi. At 14 dpi, the left lungs were subjected to histopathological analyses. **(B)** Representative histological images of the lung sections stained by hematoxylin and eosin (HE), Masson’s trichrome (MT), and picrosirius red (PR) in each experimental group. **(C)** The fibrotic severity was evaluated using the Ashcroft score in 5 different lesions obtained from each experimental group. The collagen contents of the left lung lobes in each experimental group were measured and normalized to the weight of each left lung. Data are shown as the mean ± SEM (*n* = 6). *p < 0.05 (measured by one-way ANOVA followed by Tukey’s test).

In the mouse model, following with a single intratracheal administration of BLM (3 mg/kg), interstitial inflammation and acute alveolitis accompanied by leukocyte accumulation are induced within 7 days. Patchy fibrosis is observed at 7 dpi, hereafter denoted as the early fibrotic stage. Thereafter, the fibrotic responses associated with excessive accumulation of ECM are observed. At 14 dpi, the alveolar spaces are filled with fibrous masses comprised of myofibroblasts and collagen (Yamauchi et al., 2011; Kobayashi et al., 2015). In most cases of patients diagnosed with lung fibrosis, activated myofibroblasts already harbor fibroblastic foci, which is indicative of the early fibrotic stage (King et al., 2001). To evaluate the antifibrotic effect of UNC0379 on BLM-induced lung fibrosis, we administered the mice with UNC0379 at 7, 8, and 9 dpi of BLM as it was considered a clinically practical protocol (Figure 2A).

At 14 dpi of BLM, we observed a thickening of the alveolar walls, fibrotic lesions, and an accumulation of inflammatory cells in the lung tissue samples by HE staining. Moreover, MT and PR staining revealed distinct lung fibrosis associated with collagen deposition (Figure 2B). In line with our expectations, UNC0379 efficiently ameliorated BLM-induced lung fibrosis. This finding was supported by the evaluation of the Ashcroft score and changes in the collagen content in the lung samples (Figure 2C). Interestingly, HE staining showed that the infiltrated inflammatory cells tended to remain in the alveolar space of the BLM-exposed lung tissue even after treatment with UNC0379 (insets of HE staining, Figure 2B). Next, we evaluated the changes in the inflammatory cell populations in BAL fluids from BLM-instilled mice with or without UNC0379 treatment.

**FIGURE 3:**
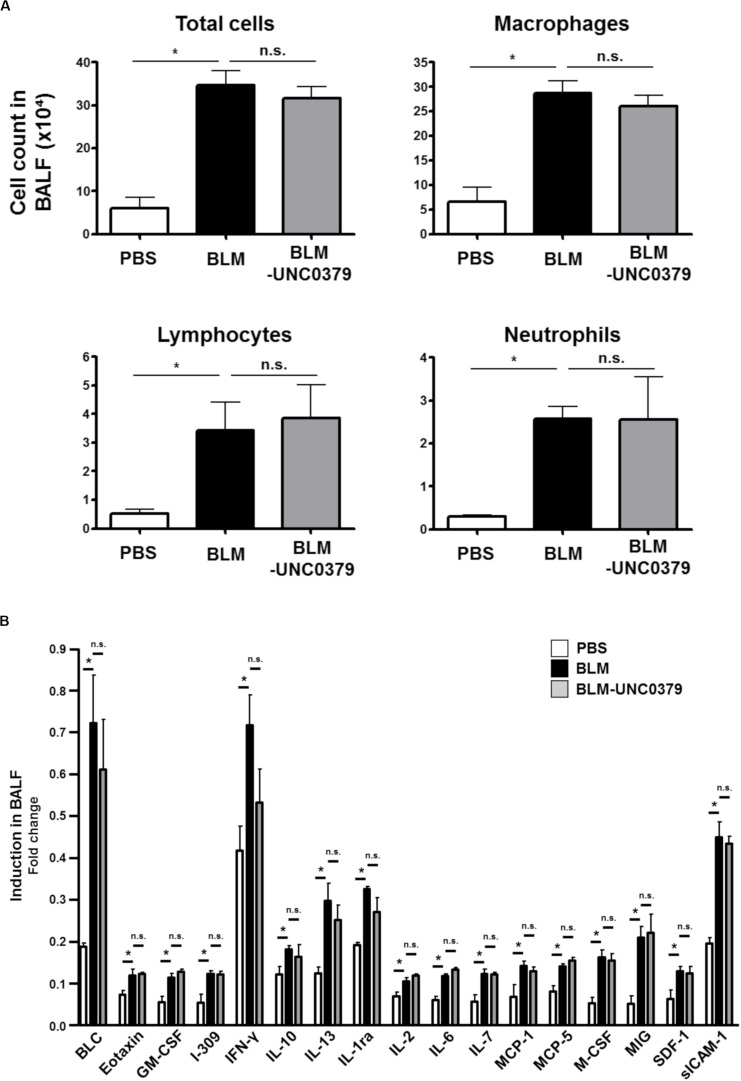
UNC0379 does not affect the inflammatory cells, but the cytokine production increased in the BAL fluids of mice instilled with BLM. **(A)** The number of total cells, macrophages, lymphocytes, and neutrophils in the BAL fluids at 10 dpi were compared between the PBS, BLM, and BLM-UNC0379 groups. Data are shown as the mean ± SEM (*n* = 4). *p < 0.05, n.s., no significant difference (measured by one-way ANOVA followed by Tukey’s test). **(B)** The expression profile of inflammatory cytokines in the BAL fluids at 10 dpi was analyzed by cytokine array and densitometric quantification. Each signal represents the mean fold-change ± SEM compared to the positive internal standard in the array membrane (*n* = 4). *p < 0.05, n.s., no significant difference (measured by one-way ANOVA followed by Tukey’s test).

As shown in Figure 3A, the number of total inflammatory cells, macrophages, lymphocytes, and neutrophils in the BAL fluids were significantly elevated at 10 dpi of BLM. The increase in these cell populations in the BAL fluids from BLM-instilled mice was not affected by treatment with UNC0379. Furthermore, a WB cytokine array of 40 cytokines showed a significant increase in the levels of 17 cytokines in the BAL fluids from mice at 10 dpi of BLM compared to the PBS-instilled control mice, and these higher levels were unaffected by UNC0379 treatment (Figure 3B). BLM-treated mice and patients with lung fibrosis often display associated pulmonary inflammation, which is characterized by the accumulation of granulocytes, macrophages, and lymphocytes (Crystal et al., 2002). These inflammatory cells are the major source of cytokines (Gharaee-Kermani and Phan, 2001). Therefore, it is reasonable to expect that the BLM-induced accumulation of inflammatory cells and the upregulation of cytokines would be equally insensitive to UNC0379. Among the BLM-upregulated 17 cytokines/chemokines, 7 molecules, namely eotaxin, interferon (IFN)-γ, interleukin (IL)-10, IL-13, IL-6, monocyte chemotactic protein (MCP)-1, and macrophage colony stimulating factor (M-CSF), were demonstrated to be profibrotic mediators in gene-knockout mice subjected to lung fibrosis induced by BLM, radiation, or silica (Chen et al., 2001; Barbarin et al., 2004; Huaux et al., 2005; Baran et al., 2007; Saito et al., 2008; Chung et al., 2016). Thus, the question arises as to why UNC0379 could effectively counter lung fibrosis, even in a lung microenvironment containing profibrotic cytokines. It is possible that the potent anti-FMD action of UNC0379 may contribute to this phenomenon. It is widely accepted that TGF-β1 is one of the most potent profibrotic factors (Fernandez and Eickelberg, 2012). Further differentiation of myofibroblasts by TGF-β1 was found to be markedly inhibited in the presence of UNC0379 (Supplementary Figure S4), suggesting that UNC0379 may bypass the action of profibrotic cytokines, at least in the BLM-induced lung fibrosis model. Taken together, these results indicate that UNC0379 effectively ameliorates BLM-induced lung fibrosis without affecting pulmonary inflammation. Based on these findings, we subsequently evaluated whether the expression and localization of SET8, the target of UNC0379, changes in response to BLM or UNC0379.

**FIGURE 4:**
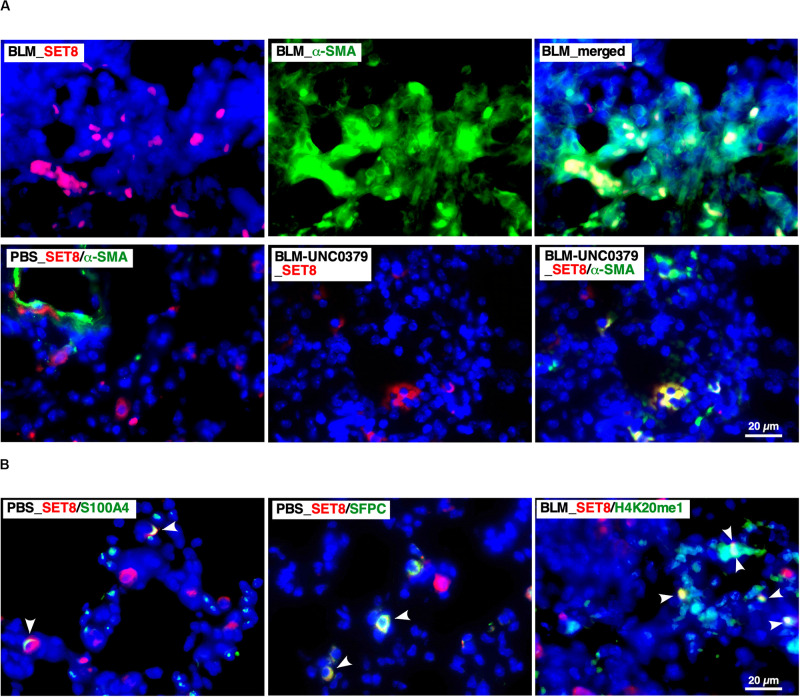
Localization of SET8 in the lung under varying conditions. **(A)** Representative images of immunofluorescence staining of SET8 (red) and α-SMA (green) in lung sections obtained from mice in the BLM, PBS, and BLM-UNC0379 groups. Cell nuclei were stained with DAPI (blue). **(B)** Representative image of the immunofluorescence staining of SET8 (red), S100A4, SFPC, and H4K20me1 (green) in lung sections obtained from the PBS and BLM groups. Arrowheads indicate cells and nuclei double positive for the Abs.

In the 14 dpi of BLM (BLM group) lung tissue samples, SET8 was typically abundant in the nucleus of α-SMA-positive cells, which formed hyperplastic lesions (Figure 4A). Several SET8-positive nuclei in the lesion contained H4K20me1 (Figure 4B). On the other hand, in the control lung tissue (PBS group), the number of cells enriched for SET8 was lower than that in the BLM group. SET8 in the PBS group was mainly observed in the cytoplasm of the parenchymal cells, such as SFPC-positive type II alveolar epithelial cells (AEC II) and S100A4-positive interstitial fibroblasts, as well as in an α-SMA-positive circular structure, which was attributed to the smooth muscle layer of lymphatic and blood vessels (Figures 4A,B). Compared to the BLM group, the frequency of the SET-8 signal was markedly reduced in the lung samples treated with UNC0379 (BLM-UNC0379 group). In the residual fibrotic area in the BLM-UNC0379 group, SET-8 localized in the cytoplasm was found to overlap with some α-SMA-positive cells (Figure 4A). The nuclear-cytoplasmic localization of SET8 has been previously demonstrated to switch during the cell cycle, in which the late SV40 factor and α-tubulin cooperatively regulate the localization and function of SET8 (Chin et al., 2020). Moreover, it has been reported that H4K20 methylation by SET8 localized to the nuclei is specifically upregulated during mitosis (Rice et al., 2002). Furthermore, in IPF fibroblasts, cell cycle regulators, such as protein regulator of cytokinesis 1 and CDC28 protein kinase regulatory subunit 1B, are upregulated, which is characteristic of aggressive cancers, and may result in the cancer-like proliferation of these cells (Emblom-Callahan et al., 2010). In the context of the previous studies, our findings suggest that the SET8/H4K20 methylation axis may be upregulated in myofibroblasts undergoing mitosis in lung tissue exposed to BLM. The administration of UNC0379 to BLM-instilled lung tissue can reverse the SET8 upregulation, as well as change the myofibroblast cell state associated with the translocation of SET8 from the nucleus to the cytoplasm. On the other hand, the fact that SET8 is expressed in interstitial fibroblasts and AEC II in the resting lung tissue is of interest, since these cells can differentiate to myofibroblasts through FMD and EMT, respectively. Provided that the inhibition of SET8 by UNC0379 also inhibits the EMT of AEC II in addition to the anti-FMD action, it is possible to interrupt the main routes of myofibroblast establishment in the lung under certain pathological conditions. Although further study is needed to confirm these findings, SET8 appears to promote EMT-associated tumorigenesis in conjunction with TWIST, a master regulator of EMT, at least in breast cancer cells (Yang et al., 2012).

The SET8-mediated monomethylation of H4K20 regulates the DNA damage repair, recruitment of DNA replication machinery, transcription, mitosis, and cell cycle progression, and can influence a broad signaling network in various cellular events, including development and pathogenesis (van Nuland and Gozani, 2016). In particular, many recent studies investigating SET8 have focused on cancer. SET8 was recently demonstrated to play a crucial role in the self-renewal, growth, and cell viability of medulloblastoma, which is the most prevalent malignant brain tumor in children (Veo et al., 2019). While studying the relationship between SET8 and cancer, it was found that SET8 can inactivate p53 via the methylation of K382 (Zhu et al., 2016). In addition, the inhibition of SET8 by UNC0379 can activate the p53-mediated proapoptotic programs in high-risk neuroblastoma (Veschi et al., 2017). One common pathological characteristic between IPF myofibroblasts and cancer cells is resistance to apoptosis, suggesting that p53-activation secondary to SET8 inhibition may specifically induce apoptosis in IPF myofibroblasts (Wynes et al., 2011). Moreover, the overexpression of p53 in lung fibroblasts can inhibit BLM-induced lung fibrosis (Nagaraja et al., 2018). In human hepatic carcinoma cell lines, UNC0379 reduced level of an inactive form of p53 (p53^K382me1^), which correlated with SET8 downregulation, and induced normal p53 expression (Wu et al., 2020). Therefore, we investigated whether p53 plays a role in the effect of UNC0379 on BLM-induced lung fibrosis. As shown in Supplementary Figure 5A, p53 was found to be localized to the nuclei of parenchymal cells in the PBS group. In the BLM group, on the other hand, p53-positive nuclei were frequently attributed to infiltrated macrophages and rarely overlapped with α-SMA-positive cells. Notably, p53-positive nuclei were typically observed along the edges of α-SMA-positive residual fibrotic lesions in the BLM-UNC0379 group. Likewise, some of the p53-positive nuclei in the margins of the residual fibrotic lesions were also positive for active caspase 3, indicating that these cells were apoptotic. As shown in Supplementary Figure 5B, p53 was markedly upregulated by UNC0379 in IPF-MyoF, which was inversely correlated with SET8 expression, supporting the localization profiles of SET8 and p53 between the BLM and BLM-UNC0379 groups (Supplementary Figure S4 and Figure 4A). These results suggest that the upregulation of p53, along with the downregulation of SET8 by UNC0379, may play an anti-fibrotic role in conjunction with UNC0379-induced dedifferentiation of myofibroblasts as well as inhibition of FMD.

## Conclusion

In the present study, the SET8 inhibitor UNC0379 was identified as a potent anti-fibrotic agent using an assay system utilizing myofibroblasts from IPF-affected lung tissue. The inhibition of SET8 by UNC0379 effectively mitigated fibrogenesis both *in vitro* and *in vivo*. To our knowledge, this study is the first to provide evidence of SET8 as a potential therapeutic target in lung fibrosis.

## Data Availability Statement

All datasets generated for this study are included in the article/Supplementary Material.

## Ethics Statement

The studies involving human participants were reviewed and approved by the Ethics Committee of Chiba University, Graduate School of Medicine and Hospital. The patients/participants provided their written informed consent to participate in this study. The animal study was reviewed and approved by the Animal Welfare Committee of Chiba University.

## Author Contributions

KU, SM, and YK developed the concept and designed the experiments. KU, SM, HM, AS, TM, and YK performed the experiments. HN, KT, MH, and TM gave conceptual advice. KU, SM, and YK wrote the manuscript. All authors discussed the results and implications and commented on the manuscript at all stages.

## Conflict of Interest

The authors declare that the research was conducted in the absence of any commercial or financial relationships that could be construed as a potential conflict of interest.

## Abbreviations

AEC II: type II alveolar epithelial cells
BAL: bronchoalveolar lavage
BLM: bleomycin
ECM: extracellular matrix
EMT: epithelial-mesenchymal transition
FMD: fibroblast to myofibroblast differentiation
FN: fibronectin
IPF: idiopathic pulmonary fibrosis
IPF-MyoF: IPF myofibroblasts
NHLF: normal human lung fibroblasts
SP-C: surfactant protein-C
α-SMA: α-smooth muscle actin
S100A4: S100 calcium binding protein A4
TGF- β: transforming growth factor- β
UIP: usual interstitial pneumonia.
