# Supplementary material for: Inhibition of the SET8 Pathway Ameliorates Lung Fibrosis Even Through Fibroblast Dedifferentiation
Source: Front Mol Biosci. 2020 Aug 5;7:192. doi: 10.3389/fmolb.2020.00192 (PMC7419601; doi:10.3389/fmolb.2020.00192)
Supplement: Supplementary file 1 [file Data_Sheet_1.pdf]

Supplementary figure 1

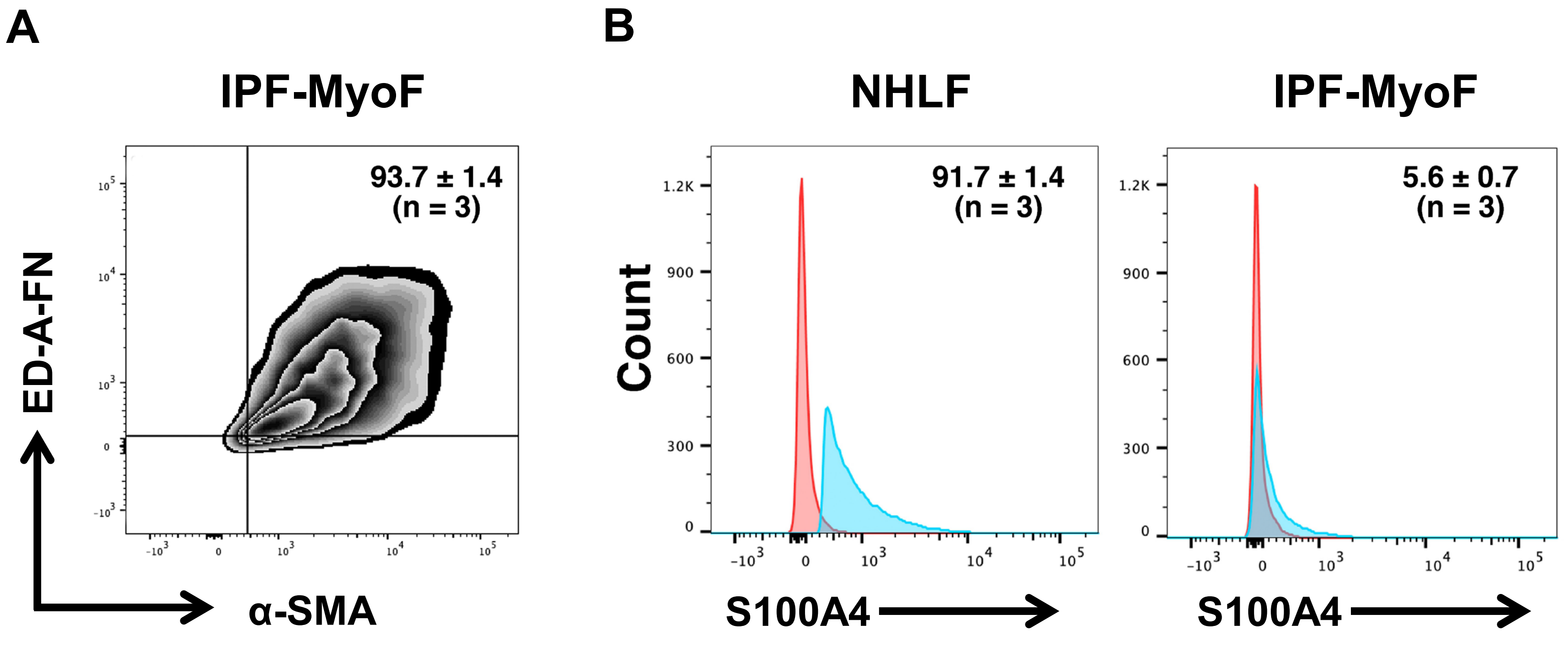

Supplementary Figure 1. Characterization of IPF-MyoF. (A) Freshly harvested IPF-MyoF were treated with 1:10 FcR blocking agent (Miltenyi Biotec) for 15 min at 4˚C. After fixing and permeabilizing (Fix/Perm buffer, Biolegend), the cells were further subjected to intracellular staining with primary antibodies (mouse anti-ED-A-fibronectin (IST-9) monoclonal antibody (sc-59825; Santa Cruz Biotech.) and rabbit anti-α-SMA polyclonal antibody (ab5694; Abcam)) for 30 min at 4˚C followed by reaction with secondary antibodies (PerCP/Cy5.5-conjugated goat anti-mouse IgG (405314; Biolegend) and Alexa Flour 488-conjugated chicken anti-rabbit IgG (A21441; Thermo Fisher Scientific)) for 15 min at 4˚C. Primary and secondary antibodies were used at a dilution ratio of 1/100 and 1/200, respectively. The resulting cells were filtered through a 70 µm-cell strainer (BD Biosciences) and analyzed with a FACSCantoII (BD Biosciences). Positively stained cells were gated using negative control cells incubated with secondary antibodies. Data were collected and analyzed with FACSDiva (BD Biosciences) and FlowJo 10.6.2 software (TreeStar, Ashland, OR). Quantitative data are shown as mean ± SEM (n = 3). **(B)** Expression level of S100A4 in NHLF and IPF-MyoF was evaluated by FAC scan according to the protocol described in **A**. Primary antibody (mouse anti-S100A4 monoclonal antibody (ab93283; Abcam)) and secondary antibody (PerCP/Cy5.5-conjugated goat anti-mouse IgG) were used. Quantitative data are shown as mean ± SEM (n = 3).

Supplementary figure 2

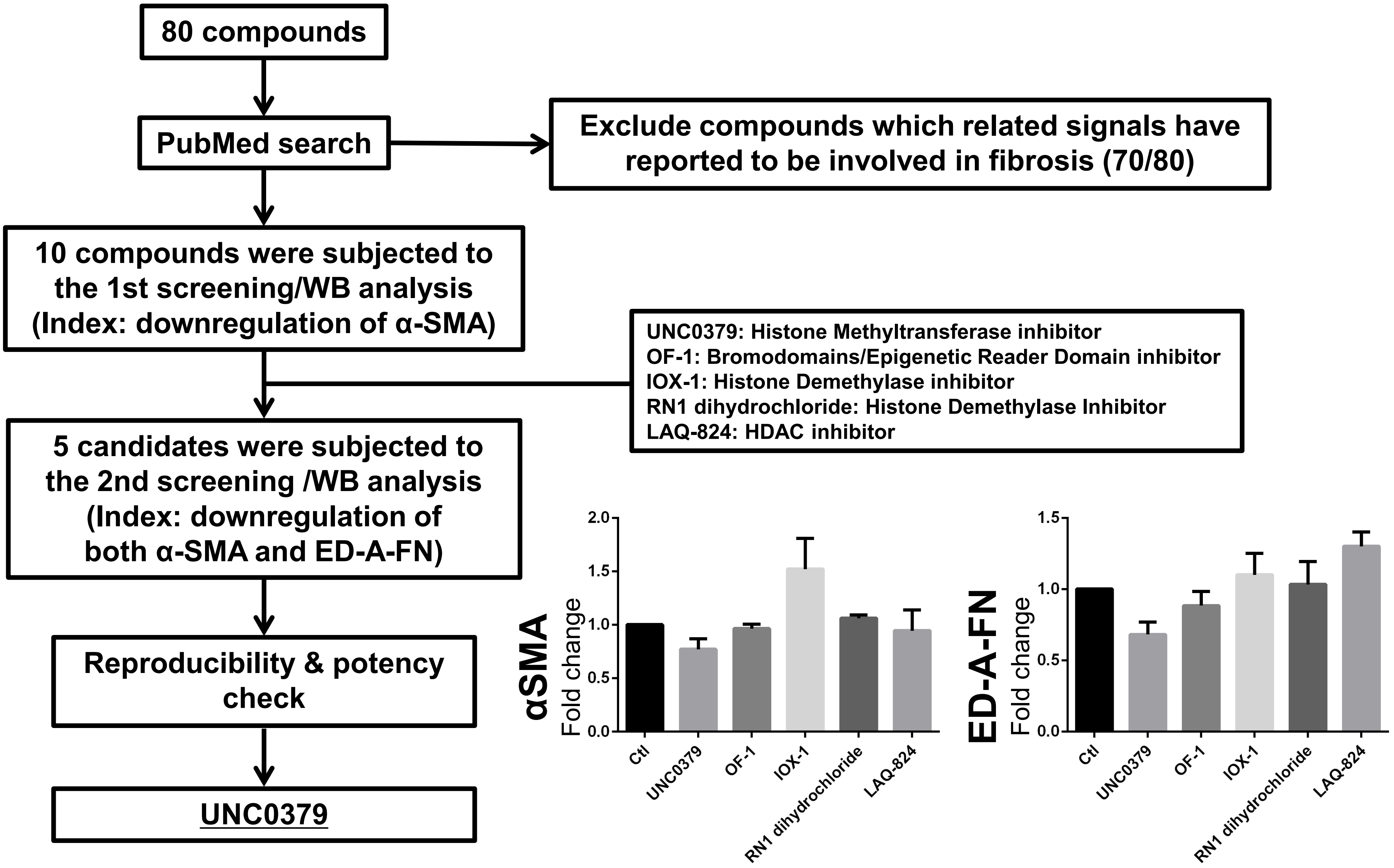

Supplementary Figure 2. Flowchart of identifying a new beneficial agent for lung fibrosis: A small library of epigenetics-related chemical compounds (80 compounds, Sigma-Aldrich, St.Louis, MO, USA) was purchased, and they and their target molecules were systematically reviewed from the view point of correlation with fibrosis. Selected 10 compounds having no report regarding fibrosis were subjected to the 1st screening by Western blotting (WB) to assess downregulation of α-SMA in established myofibroblasts from the IPF lung tissue. After that, 5 candidates were subjected to the 2nd screening to validate downregulation of α-SMA and ED-A-FN in the IPF-MyoF. After examining reproducibility and potency, UNC0379 was identified as the new potential therapeutic agent that can induce dedifferentiation of the IPF-MyoF. The expression levels of α-SMA and ED-A-FN in IPF-MyoF treated with each candidate compound were normalized for β-actin by a densitometer. All quantitative data are presented as mean ± SEM (n=3).

Supplementary figure 3

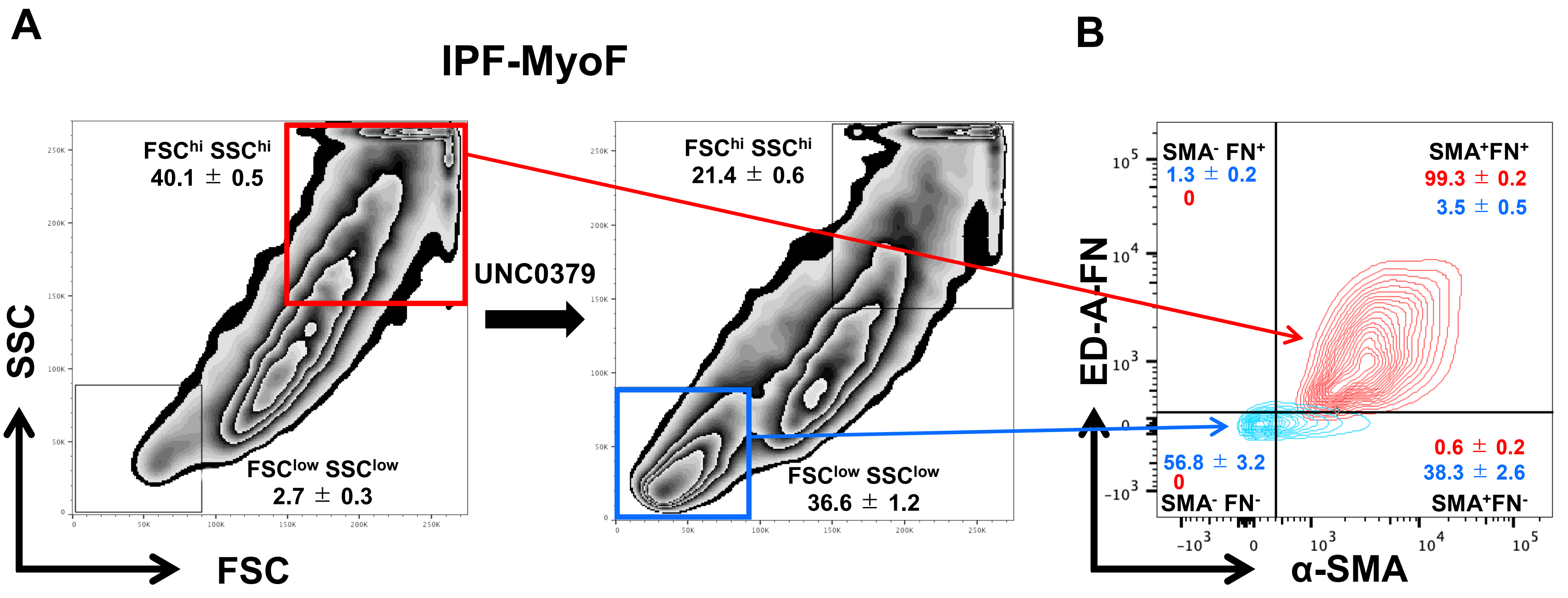

Supplementary Figure 3. Phenotypic changes of IPF-MyoF by treatment with UNC0379. Freshly harvested IPF-MyoF with or without UNC0379 treatment (10 µM for 48 h) were subjected to intracellular staining of ED-A-FN and α-SMA. The procedure and data analysis were performed according to those described in Supplementary figure 1. Quantitative data are shown as mean ± SEM (n = 3). **(A)** IPF-MyoF with or without UNC0379 treatment were plotted as side scatter (SSC) versus forward scatter (FSC). IPF-MyoF fell into the $FSC^{hi}SSC^{hi}$ gate, which was significantly reduced by the treatment with UNC0379 (40.1 ± 0.5% *vs* 21.4 ± 0.6%, $p < 0.0001$, unpaired *t*-test). In contrast, the $FSC^{low}SSC^{low}$ population of IPF-MyoF significantly increased in response to UNC0379 (2.7 ± 0.3% *vs* 36.6 ± 1.2%, $p < 0.0001$, unpaired *t*-test). These data suggest that cell size and intracellular organelle complexity decrease in the UNC0379-treated IPF-MyoF. **(B)** Fluorescent intensity for ED-A-FN and α-SMA was evaluated in the $FSC^{hi}SSC^{hi}$ population of IPF-MyoF (red contour plots) and the $FSC^{low}SSC^{low}$ population of the UNC0379-treated IPF-MyoF (light blue contour plots). The cell populations were divided into four fractions (α-SMA^+FN^+; α-SMA^+FN^-; α-SMA^-FN^-; α-SMA^-FN^+) based on the expression pattern of α-SMA and ED-A-FN. The proportion of each fraction as a percentage of total cells is shown.

Almost all $FSC^{hi}SSC^{hi}$ population of IPF-MyoF was α-SMA^+ED-A-FN^+(99.3 ± 0.2%), indicating that the $FSC^{hi}SSC^{hi}$ population was apparently positive to the two fibrotic markers. In contrast, α-SMA^+ED-A-FN^+ fraction was negligibly observed in the $FSC^{low}SSC^{low}$ population of the UNC0379-treated IPF-MyoF (3.5 ± 0.5%). In the $FSC^{low}SSC^{low}$ population (light blue contour plots), a single positive fraction to α-SMA was approximately 42%, the fluorescent intensity of which was lower than that of the $FSC^{hi}SSC^{hi}$ population (red contour plots). Likewise, a single positive fraction to ED-A-FN in the $FSC^{low}SSC^{low}$ population was approximately 5% and showed dim fluorescent intensity. Hence, the $FSC^{low}SSC^{low}$ population was composed of four fractions, α-SMA^{low}ED-A-Fn^{dim} (3.5 ± 0.5%), α-SMA^{low}ED-A-Fn^- (38.3 ± 2.6%), α-SMA^-ED-A-Fn^- (56.8 ± 3.2%) and α-SMA^-ED-A-Fn^{dim} (1.3 ± 0.2%) Taken together, UNC0379-reduced cell size and intracellular organelle complexity was correlated with a decrease in expression of the two fibrotic markers.

Incidentally, 99.4% of the $FSC^{hi}SSC^{hi}$ population in the UNC0379-treated IPF-MyoF were α-SMA^+ED-A-FN^+, and 2.7% of the $FSC^{low}SSC^{low}$ population in the IPF-MyoF were α-SMA^+ED-A-$FN^{+(dim)}$.

Supplementary figure 4

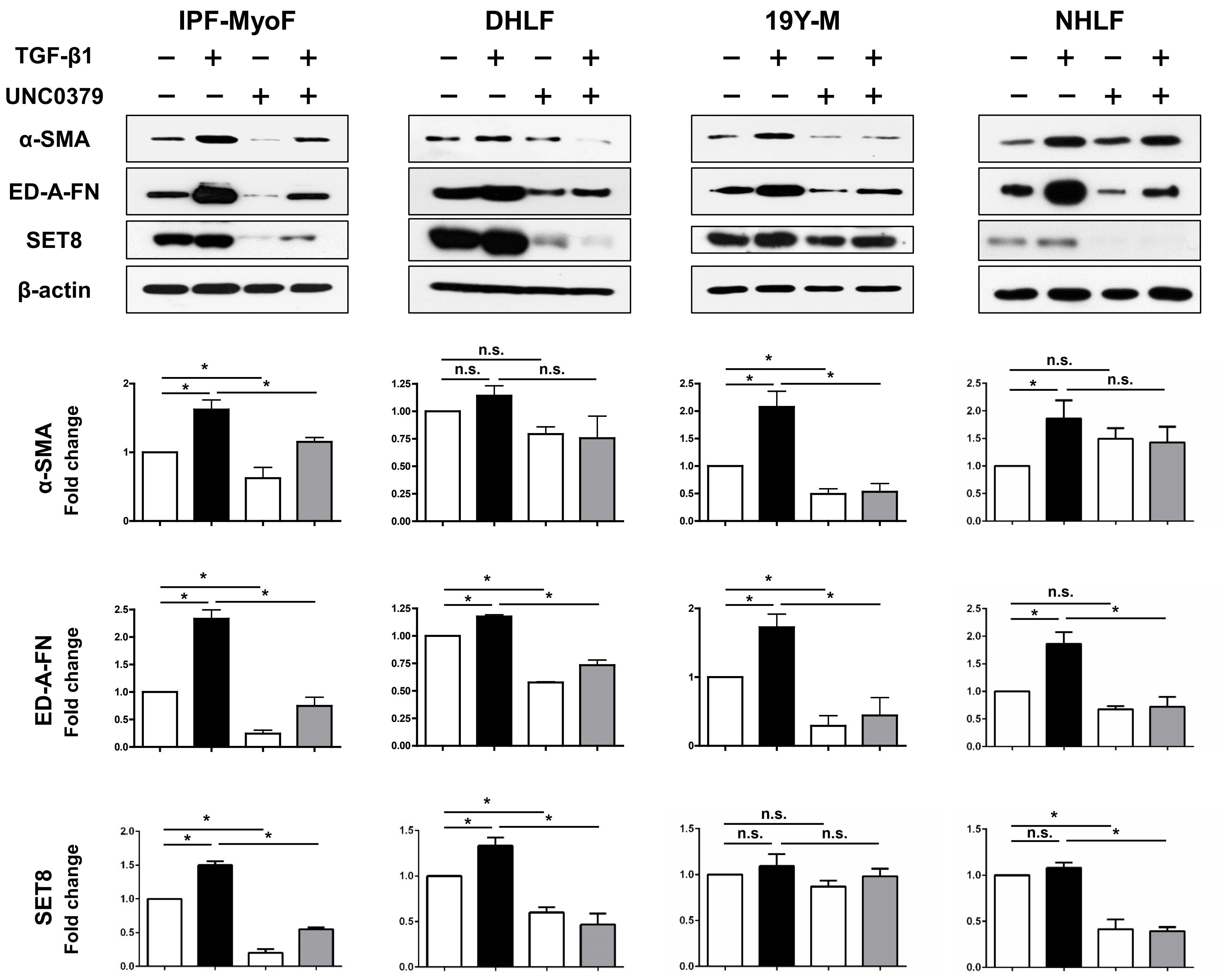

Supplementary Figure 4. UNC0379 induced downregulation of fibrotic marker proteins and inhibited FMD stimulated with TGF-β1 in three lines of primary cultured lung myofibroblasts with different clinical background (IPF-MyoF, DHLF and 19Y-M) and normal human lung fibroblast (NHLF). All kinds of cells were treated with 10 μM UNC0379 or/and 10 ng/ml TGF-β1 for 48 h. The expression levels of α-SMA, ED-A-FN, SET8 and β-actin (internal control for normalization) were evaluated by WB. All quantitative data are shown as mean fold change ± SEM compared to the control value of untreated cells (n = 3). **P*<0.05, n.s., no significant difference (measured by one-way ANOVA followed by Tukey's test).

Antibodies were used as follows: rabbit anti-α-SMA polyclonal antibody (ab5694; Abcam), mouse anti-FN antibody (sc-59825, clone IST-9, ED-A domain-specific; Santa Cruz Biotech.), rabbit anti-SET8 Ab (2996; Cell Signaling Technology), and mouse anti-β-actin Ab (A2228, clone AC-15; Sigma-Aldrich).

Supplementary figure 5

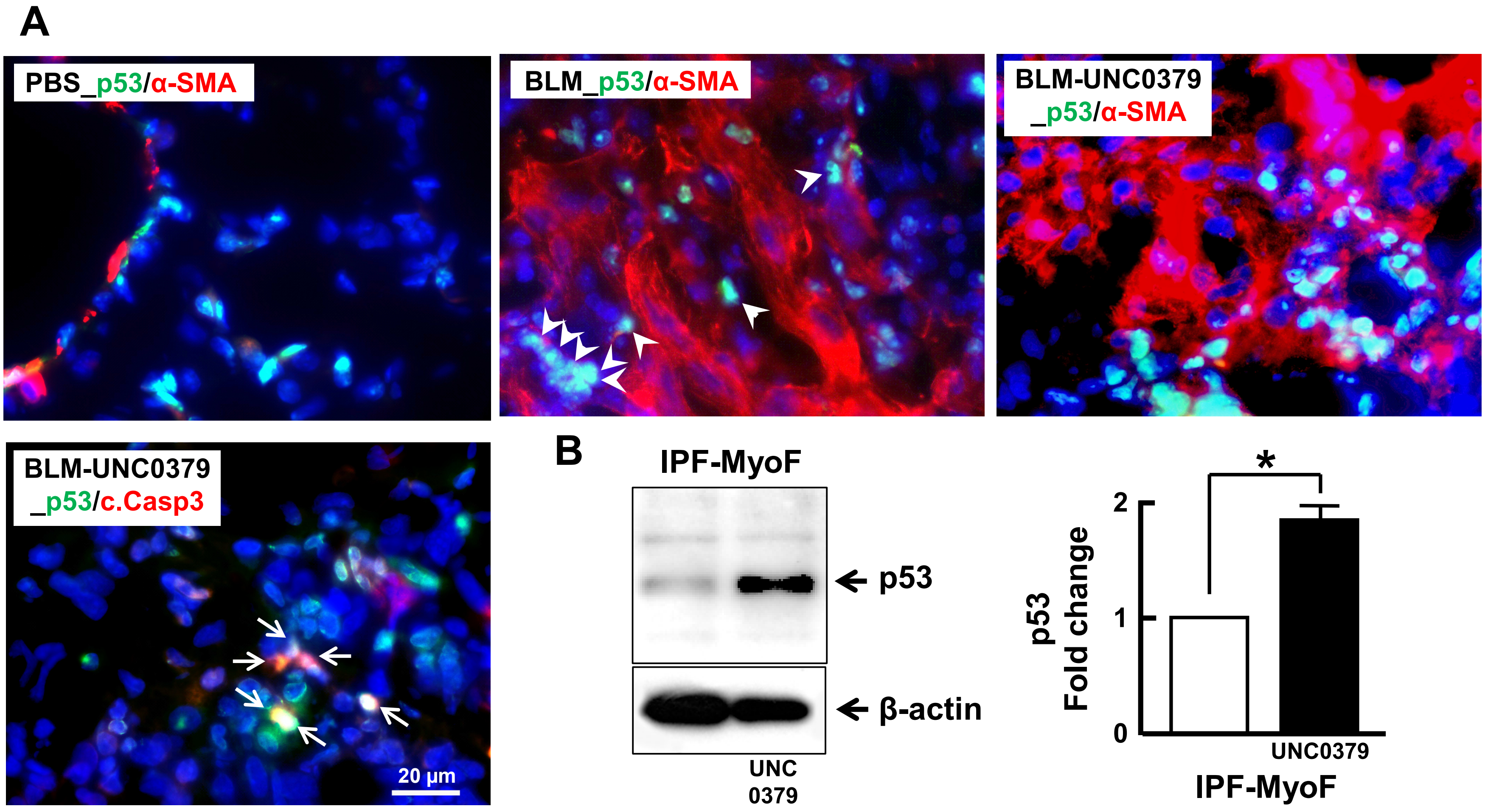

Supplementary Figure 5. UNC0379 induced upregulation of p53. (A) Representative images of immunofluorescence staining for p53 (green) and α-SMA (red) in lung sections obtained from mice of PBS, BLM, and BLM-UNC0379 groups at 14 dpi and for p53 (green) and cleaved caspase 3 (c.Casp3) (red) in lung section obtained from mice of BLM-UNC0379 group at 14 dpi. Cell nuclei were stained with DAPI (blue). Arrow heads indicate nuclei of infiltrated macrophages. Arrows indicate $p53^{+}c.Casp3^{+}$ nuclei. Notably, p53-positive nuclei are typically observed in the marginal area of residual fibrotic lesion in the BLM-UNC0379 group. Some of the p53-positive nuclei are also positive for cleaved caspase 3, indicating apoptotic cell state. **(B)** IPF-MyoF were treated with 10 μM for 48 h. The expression levels of p53 and β-actin (internal control for normalization) were evaluated by WB. Quantitative data are shown as mean fold change ± SEM compared to the control value of untreated cells (n = 3). *$P<0.05$, significant difference (measured by unpaired *t*-test). Dedifferentiation of IPF-MyoF induced by UNC0379 may be associated with upregulation of p53.

Antibodies were used as follows: mouse anti-p53 (DO-1) monoclonal antibody (SC-126; Santa Cruz Biotech.); rabbit anti-α-SMA polyclonal antibody (ab5694; Abcam); rabbit anti-cleaved caspase 3 (Asp175) (5A1E) monoclonal antibody (9664; Cell Signaling Tech.) and mouse anti-β-actin monoclonal antibody (A5441; Sigma-Aldrich).
